# Extraction, Purification, Component Analysis and Bioactivity of Polyphenols from *Artemisia dracunculus* L.

**DOI:** 10.3390/foods14101823

**Published:** 2025-05-21

**Authors:** Lin Chen, Buhailiqiemu Abudureheman, Omar Anwar, Emran Abdugini, Jianlin Zhang, Rui Tang, Zhihui Gao, Haibo Pan, Xingqian Ye

**Affiliations:** 1 Center for Experimental Instruction in Food Safety and Nutrition, Xinjiang Institute of Technology, Aksu 843000, China; nei3677585@163.com (L.C.); 17690041371@163.com (O.A.); 17609004281@163.com (E.A.); zjl950702@163.com (J.Z.); 15309973690@189.cn (R.T.); 19590112684@163.com (Z.G.); 2 College of Biosystems Engineering and Food Science, Zhejiang University, Hangzhou 310058, China; psu@zju.edu.cn; 3 Innovation Center of Yangtze River Delta, Zhejiang University, Jiaxing 314102, China

**Keywords:** *A. dracunculus* L., extraction and purification, component analysis, antioxidant capacity, antibacterial capacity

## Abstract

*A. dracunculus* L., is a species of traditional Chinese medicine herbs, widely distributed northwestern China and used as antidiabetic, antibacterial etc., but the active compounds and their abundance have not been systematically investigated. This research focused on the following: (i) optimizing polyphenol extraction/purification from *A. dracunculus*; (ii) UPLC-QE-based profiling of polyphenolic composition; (iii) FT-IR-assisted structural elucidation; and (iv) functional assessment of antioxidant and antibacterial properties. The results showed that the highest extraction yield of crude polyphenols of *A. dracunculus* (CPA) reached 5.02 ± 0.04% at an ethanol concentration of 70% of 70 °C with a solid-to-liquid ratio of 1:20 (g/mL). The D101 macroporous resin is the best one for polyphenolpurification of *A. dracunculus* (PPA), with a purification efficiency of 60.48 ± 1.87%. UPLC-QE analysis identified 36 polyphenolic compounds in PPA, in whic the content of protocatechuic acid is the highest at 1338.05 ± 1.83 ng/mg. The absorption peaks at 1691 cm^−1^ (carbonyl, C=O), 1605 cm^−1^and 1518 cm^−1^ (aromatic C=C), as well as 1275 cm^−1^ and 1369 cm^−1^ (C-O stretching), indicated the presence of phenolic acids, flavonoids and tannins in PPA by FT-IR. PPA exhibited significant antioxidant activity, which reached 81.73 ± 1.43% for DPPH, 87.11 ± 1.57% for hydroxyl and 85.74 ± 1.52% for ABTS+. It also demonstrated strong antibacterial activity against nine common pathogenic bacteria, but not to Escherichia coli. *A. dracunculus* polyphenols demonstrate potent bioactive properties, suggesting potential applications in functional foods and natural preservatives.

## 1. Introduction

*Artemisia dracunculus* L. is primarily distributed in northern and northwestern China, where its entire plant is utilized for both medicinal and culinary purposes [1]^.^ *A. dracunculus* exhibits a range of therapeutic properties, including heat clearing, wind dispelling, antiviral [2], antioxidant [3], and immune-enhancing effects [4]. It is traditionally used to treat colds caused by wind-cold, edema, and scurvy [5]. *A. dracunculus* also has a long history of use in food preparation, commonly employed as a spice in daily cooking and used as a flavoring agent in salads, meat products, beverages and alcoholic drinks [6].

Research has identified various bioactive compounds in *A. dracunculus*, including polysaccharides, lactones, coumarins, organic acids, flavonoids, sterols, triterpenoids, and alkaloids [7,8]. Phenolic compounds are a class of secondary metabolites, characterized by at least two phenyl rings and one or more hydroxyl substituents [9], widely present in plants such as fruits, vegetables, cereals, tea, coffee, etc., in which, the polyphenols mainly exist in the form of glycosides. Polyphenols can be simply classified into flavonoids and nonflavonoids, and subdivided into many subclasses depending on the number of phenol units, such as the flavonoids, including flavanols, anthocyanidins, anthocyanins, isoflavones, flavones, flavonols, flavanones, and flavanonols [10]. Polyphenolic compounds possessing diverse health beneficial properties such as antioxidant and antibacteria and fungi anti-inflammatory and play a significant role in the prevention of chronic diseases such as cardiovascular diseases, diabetes, obesity, and neurodegenerative diseases [11,12]. Different phenolic components exhibit distinct physiological functions, making the identification of plant polyphenolic components crucial for understanding their physiological roles and medicinal value [13]. Reactive oxygen species (ROS) are required for normal physiological metabolism in humans. Polyphenols have been found to modulate these pathways effectively, functioning as dietary antioxidants [14].

Polyphenolic compounds are typically isolated through various extraction methodologies such as supercritical and organic solvent-based techniques, sonication-assisted processes, microwave-mediated approaches, ionic precipitation and biocatalytic extraction. Supercritical fluid extraction (SFE) offers an environmentally friendly alternative to conventional extraction methods as it eliminates the need for organic solvents. By carefully controlling temperature and pressure parameters, SFE allows for selective recovery of heat-sensitive polyphenolic compounds while maintaining their structural integrity [15]. Ultrasound-assisted extraction (UAE) enhances extraction efficiency through ultrasonic cavitation, which accelerates mass transfer while reducing processing time [16]. Enzymatic extraction utilizes cellulase or pectinase to hydrolyze plant cell walls, enhancing polyphenol release. This biological method offers superior selectivity and preserves compound stability under mild processing conditions [17] (Gao et al., 2022). However, the methods of SFE, UAE and enzymatic extraction have disadvantages, including high costs, which lead to polyphenol degradation and complication of the process; solvent extraction is the most simple and low-cost method and is the most widely adopted method [18,19]. Resin-based separation and purification techniques have gained significant research prominence owing to their operational simplicity and economic viability in various applications [20]. Macroporous adsorption resins have high adsorption capacity, rapid adsorption rates, high selectivity [21]. The combination of solvent extraction and macroporous resin adsorption is the most efficient and economical way to purify polyphenolic compounds [22].

Current studies on *A. dracunculus* primarily focus on its essential oils and pharmacological properties [2]. Research on *A. dracunculus* polyphenolic compounds holds significant potential for enriching the natural medicinal resource library [23]. However, there are no reports about the structural analysis and biological activities of purified polyphenols of *A. dracunculus* (PPA). In this research, the solvent extraction condition and purification methods of polyphenol extract of *A. dracunculus* were determined, the polyphenolic compounds analyzed and confirmed the structure of PPA. Also, the capacity of antioxidant and antibacterial were measured. This research provides a theoretical foundation for the effective utilization of wild medicinal and edible plant resources.

## 2. Materials and Methods

### 2.1. Materials and Chemicals

The samples of *A. dracunculus* (a mixture of leaves and stems) were harvested from the Tomur Peak Nature Reserve of southern foothills in the Tianshan Mountains (Aksu, China), which were stored at 4 °C. All the standards were products of Sigma Chemicals Co. (St. Louis, MO, USA). AB-8, D-101, D-201, D-301, and NKA-9 macroporous resins were purchased from Shanghai Yuanye Biotechnology Co. (Shanghai, China) (Table 1). Ethanol and other chemicals were purchased from Sinopharm Chemical Reagent Co. (Shanghai, China). *Salmonella enterica*, *Staphylococcus aureus*, *Bacillus subtilis*, *Escherichia coli*, *Shigella flexneri*, *Enterococcus faecalis*, *Pseudomonas aeruginosa*, *Listeria monocytogenes*, *Acinetobacter baumannii*, and *Rhizopus stolonifer* were purchased from the China Center for Industrial Culture Collection (CICC, Beijing, China). All other chemicals and reagents used in our study were of analytical and chromatographic grade.

### 2.2. Polyphenol Extraction Methods

The powdered sample of *A. dracunculus* with the moisture content of 5.48 ± 0.33% was sieved (sieve aperture: 0.355 mm, mesh size: 45 mesh) and extracted under different extraction temperatures (40, 50, 60, 70, 80 °C), solid-to-liquid ratio (1:10, 1:15, 1:20, 1:25, 1:30, g/mL) for 30, 45, 60, 75, 90 min by ethanol concentrations at 100%, 90%, 80%, 70%, 60% using a water bath, for three times separately, to investigate the effects of different conditions on extraction yield of crude polyphenols of *A. dracunculus* (CPA). After this, the extracts were concentrated at rotary evaporator and centrifuged at 6000× *g* for 10 min, and freeze-dried [24].

Based on preliminary single-factor experiments, three independent variables were designed using a three-factor Box-Behnken design (BBD), including ethanol concentration (A, 70%, 80%, 90%), solid-to-liquid ratio (B, 20, 30 and 40, g/mL) and extraction temperature (C, 50 °C, 60 °C, and 70 °C). The polyphenols yields were used as response-dependent values [25]. All experimental procedures were performed in triplicate. The coded values and corresponding operational ranges for different parameters are shown in Table 2.

### 2.3. Static Adsorption and Desorption Experiments on CPA

Adsorption method: 5 g of each pretreated macroporous resin (D301, D201, D101, NKA-9 and AB-8) were weighed and combined with 20 mL of the CPA solution at a concentration of 5 mg/mL, and oscillated for 4 h at adsorption conditions of 25 °C and 190 r/min in the dark [26]. Then, the polyphenol concentration in the filtrate was measured, and the adsorption capacity and adsorption rate were calculated using Equations (1) and (2).

Desorption method: the macroporous resin was subjected to 30 mL of 70% (*v*/*v*) ethanol solution for surface washing after filtration, and this solution was oscillated for 4 h under the same conditions to achieve complete desorption [27]. Finally, the desorption rate of polyphenol was measured using Equation (3).

(1) $$\mathrm{Adsorption}\;\mathrm{capacity}(\mathrm{mg}/g)=\frac{{(C}_{0}-C_{1})V_{1}}{m}$$

(2) $$Adsorption\;rate(\%)=\frac{\left(C_{0}-C_{1}\right)}{C_{0}}\times 100$$

(3) $$Desorption\;rate(\%)=\frac{\left(C_{2}\times V_{2}\right)}{mQ}\times 100$$

Here, the mathematical model incorporated the following parameters: *C*_0_ denoted initial polyphenolic concentration in the extract (mg/mL), *C*_1_ represented residual concentration post-adsorption (mg/mL), and *C*_2_ indicated eluted polyphenolic concentration (mg/mL). System volumes were defined as *V*_1_ for initial extract volume (mL) and *V*_2_ for eluent volume (mL); m represents wet resin quality (g) and *Q* represents adsorption capacity of the resin, expressed in mg/g.

### 2.4. Determination of Polyphenol Purification Process

The determination of characteristic curves for static adsorption kinetics are as follows: D101 macroporous resin was selected as suitable for purification of CPA, based on the result of Section 2.3. Firstly, a total of 5.0 g activated D101 resin mixed with 20 mL of 5 mg/mL CPA. Then, it was adsorbed using the method in Section 2.3, and the supernatant was sampled at every 20 min to measure the polyphenol content [27].

Determination of loading concentration: five portions of 5.00 g activated D101 macroporous resin were mixed with 20 mL of CPA at concentrations of 3.0 mg/mL, 4.0 mg/mL, 5.0 mg/mL, 6.0 mg/mL, and 7.0 mg/mL, separately. They were then adsorbed using the method in Section 2.3, and the adsorption capacity and adsorption rate were calculated using Equations (1) and (2).

Determination of eluent concentration: six portions of 5.00 g activated D101 macroporous resin were mixed with 20 mL of 5.0 mg/mL CPA, separately. Then, the adsorption and desorption method was the same as Section 2.3. However, during the desorption, 30 mL of ethanol at concentrations of 40%, 50%, 60%, 70%, 80%, and 90% were used, respectively, to evaluate its surface washing ability.

### 2.5. Dynamic Adsorption and Desorption of D101 Macroporous Resin for CPA

The activated D101 macroporous resin was wet packed into a column (Φ4.6 cm × 45.7 cm). The volume of the column bed was 3/5 of total column height and 10 mL of eluent was in one tube. A polyphenol stock solution (5 mg/mL) was prepared by dissolving 100 mg of CPA sample, followed by filtration through a 0.22 µm organic membrane. Chromatographic separation was performed at ambient temperature with a flow rate of 2 mL/min, maintained by a peristaltic pump employing rotating rollers to ensure consistent flow through flexible tubing. Following the adsorption phase, the stationary phase was rinsed with deionized water to eliminate hydrophilic impurities. Subsequent elution was conducted using 70% ethanol at a flow rate of 1.8 mL/min [28]. Polyphenolic content in the eluate was quantified through a Folin-Ciocalteu assay: a gallic acid standard (10.0 ± 0.1 mg) was quantitatively transferred to a 100 mL volumetric flask and brought to volume with deionized water, yielding a 0.1 mg/mL stock solution; six working standards were prepared by transferring 100, 200, 300, 400, 500, and 600 µL aliquots of the stock solution into separate 10 mL volumetric flasks with 1.0 mL of freshly prepared Folin-Ciocalteu reagent and 2.0 mL of 15% (w/v) sodium carbonate solution. After thorough vortex mixing (30 s), the reaction mixtures were protected from light and maintained at 25 ± 0.5 °C for 120 ± 5 min to ensure complete chromophore formation. An analytical curve was constructed utilizing a standard curve of gallic acid (Y = 04177x + 0.1048, R^2^ = 0.9975), enabling the construction of an elution profile. Fractions exhibiting elevated polyphenolic concentrations were subjected to ethanol evaporation and subsequent lyophilization to yield purified polyphenol extract (PPA). The purity assessment was based on the mass ratio of polyphenolic compounds to total extract, with polyphenolic content determined spectrophotometrically and total mass measured gravimetrically [25].

### 2.6. Component Analysis of PPA

#### 2.6.1. Sample Preparation and Extraction

The samples were subjected to extraction using 0.5 mL of 80% methanol solution supplemented with 0.2% VC. The extraction process involved ultrasonication for 30 min, followed by centrifugation at 12,000 rpm for 10 min to obtain the supernatant. This extraction procedure was repeated three times, and the resulting supernatants were pooled for subsequent analysis [29].

#### 2.6.2. UPLC Conditions

The sample extracts were subjected to chromatographic separation and mass spectrometric detection using a UPLC-Orbitrap-MS platform (Vanquish UPLC coupled with QE mass spectrometer). Chromatographic separation was performed on a Waters HSS T3 column (50 × 2.1 mm, 1.8 μm) maintained at 40 °C, with a mobile phase flow rate of 0.3 mL/min. The injection volume was set at 2 μL. A binary solvent system consisting of water and acetonitrile, each containing 0.1% formic acid, was employed with the following gradient elution profile: isocratic at 90:10 (V/V) from 0 to 2.0 min, linear transition to 40:60 (V/V) at 6.0 min, maintained until 9.0 min, then returned to initial conditions at 9.1 min and held until 12.0 min [30].

#### 2.6.3. MS Analysis

High-resolution mass (HRMS) spectrometric analysis was performed using a Q Exactive hybrid quadrupole-Orbitrap mass spectrometer (Thermo Fisher Scientific, Waltham, MA, USA) equipped with a heated electrospray ionization source. The instrument was operated in Fullms-ms^2^ acquisition mode with the following ionization parameters: negative ionization mode at 2.8 kV, sheath gas flow rate of 40 arbitrary units, auxiliary gas flow of 10 arbitrary units, ion transfer capillary maintained at 320 °C, and auxiliary gas heater set to 350 °C. Mass spectral data acquisition and processing were conducted using Xcalibur 4.1 and TraceFinder™ 4.1 Clinical software packages (Thermo Fisher Scientific), respectively, with quantitative results exported in Excel format for further analysis [31].

### 2.7. Infrared Spectroscopy (IR) Analysis

The FT-IR analysis of PPA was performed using a TENSOR 37 FT-IR spectrophotometer (Bruker, Ettlingen, Germany); the dry sample of PPA (2 mg) was mixed with an appropriate amount of KBr into tablets, and the spectral scanning was performed at the range of 400–4000 cm^−1^, with the resolution set at 2 cm^−1^ and a total of 32 scans being performed [32].

### 2.8. In Vitro Antioxidant Activity

#### 2.8.1. DPPH Radical Scavenging Activity

CPA and PPA solutions of 1 mL were placed in test tubes with concentrations of 0, 10, 20, 30, 40, 50, 60, 70, 80, 90 and 100 ug/mL, respectively. Then, 1 mL of DPPH-ethanol solution (0.2 mmol/L) was added. The tubes were placed in the dark for 30 min and their absorbance at 517 nm was measured by Abudureheman et al. [33]. The DPPH radical scavenging activity was determined by using Equation (4) and VC was used as a positive control. Results were expressed as DPPH radical scavenging activity %.

(4) $$DPPH\;radical\;scavenging\;activity(\%)=\frac{A\;control-A\;sample}{A\;control}\times 100\%$$

#### 2.8.2. Assay of ABTS^+^ Radical Scavenging Activity

CPA and PPA solutions of 0.2 mL were placed in test tubes with concentrations of 0, 10, 20, 30, 40, 50, 60, 70, 80, 90 and 100 ug/mL, respectively. ABTS^+^ radical solution was adjusted with methanol to an absorbance of 0.700 ± 0.020 at 734 nm. Then 0.8 µL of this solution of ABTS^+^ was into the tube to react for 6 min in the dark. The antioxidant capacity against cationic radicals was assessed through ABTS^+^ decolorization assay following the experimental protocol developed by Hu et al. [34] at 734 nm. The ABTS^+^ radical scavenging ability was calculated using Equation (5) and VC was used as the positive control. Results were expressed as ABTS^+^ radical scavenging activity %.

(5) $${ABTS}^{+}radical\;scavenging\;activity(\%)=[1-\frac{A\;sample-A\;control}{A\;blank}]\times 100\%$$

#### 2.8.3. The Scavenging Ability of Hydroxyl Radicals

For hydroxyl radical scavenging capacity assessment, an equal-volume reaction system was prepared containing salicylic acid (1 mL), test sample (1 mL), ferrous sulfate solution (1 mL), and hydrogen peroxide (1 mL). Aliquots (200 μL) of the reaction mixture were transferred to ELISA plates for spectrophotometric quantification at 510 nm using a microplate reader (DNM-9602G, Beijing Prolong New Technology). The radical scavenging efficiency (%) was determined according to the computational model described in Equation (6).

(6) $$Hydroxyl\;radical\;scavenging\;ability(\%)=(1-\frac{A_{1}-A_{2}}{A_{3}}\times 100\%)$$

### 2.9. In Vitro Antibacterial and Antifungal Activity

#### 2.9.1. Determination of Antibacterial and Antifungal Activity

The antimicrobial efficacy was evaluated through a modified disk diffusion assay according to the methodology established by Ngamsurach [35]. A blank agar plate was evenly divided into three regions and labeled. A volume of 100 μL of bacterial suspension of *S. enterica*, *S. aureus*, *B. subtilis*, *E. coli*, *S. flexneri*, *E. faecalis*, *P. aeruginosa*, *L. monocytogenes*, *A. baumannii*, and *R. stolonifer* was spread uniformly onto the surface of the blank plate. Then, a filter paper disc which completely absorbed the 20 μL of PPA extracts with different concentrations was placed onto the agar plate. The culture plates were subjected to 37 °C, 24 h, and the growth of the bacterial colonies was observed, and the diameter of the inhibition zones was measured.

#### 2.9.2. Determination of Minimum Inhibitory Concentration (MIC) and Minimum Bactericidal Concentration (MBC)

The minimum inhibitory concentration (MIC) of PPA against selected bacterial strains was determined using the twofold serial dilution method in a 96-well microplate. A volume of 100 μL of polyphenol extract with different concentrations was mixed with 100 μL of the bacterial suspension in the logarithmic growth phase in the 96-well microplate. The mixture was incubated at 37 °C for 24 h. Then, the MIC was determined by visual observation of turbidity and measured at an absorbance of 700 nm using a microplate reader (DNM-9602G, Beijing Prolong New Technology, Beijing, China). The highest dilution of the test solution that showed no turbidity and had an absorbance value similar to the blank control was recorded as the MIC [36].

The agar plate method was used to determine the minimum bactericidal concentration (MBC). The mixture that completely inhibited the bacterial growth was transferred to nutrient agar medium and incubated at 37 °C for 24 h. The minimum bactericidal concentration was determined as the lowest test compound dilution that maintained complete suppression of microbial proliferation on solid culture media [37].

### 2.10. Statistical Analysis

Experimental results were presented as mean values with standard deviation (mean ± SD), calculated from triplicates. Statistical evaluation of Box-Behnken design responses was performed through variance decomposition analysis utilizing Design Expert 13.0 (Stat-Ease Inc., Minneapolis, MN, USA). Figures were drawn using Origin 2021 (OriginLab Corp., Northampton, MA, USA), with *p* < 0.05 considered statistically meaningful.

## 3. Results

### 3.1. The Effect of Extraction Condition to the CPA Yield

**Figure 1:**
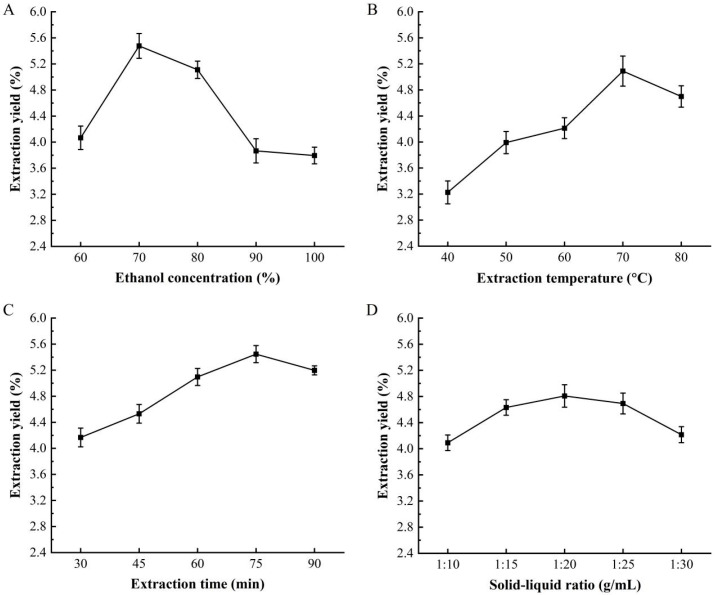
Effect of ethanol concentration (**A**), extraction temperature (**B**), extraction time (**C**), and the ratio of solid to liquid (**D**) on the yield of CPA.

The extraction yield of CPA at different conditions shown in Figure 1.The extraction yield increased with ethanol concentration from 60% to 70%; the maximum CPA extraction yield was reached at 5.48 ± 0.19% at 70% ethanol concentration. However, the extraction yield of CPA decreased when the ethanol concentration exceeded 70% (Figure 1A). Alara [38] also found that the optimal extraction parameters for polyphenols from *Vernonia cinerea* leaves was 60% ethanol concentration achieving a crude polyphenol yield of (10.01 ± 0.85)% (*w/w*) at 60 °C for 2 h of extraction time and the CPA yield decreased to 8.03 ± 0.90% (*w/w*) at 80% ethanol concentration. This decline may be due to the increased polarity gap between polyphenols and the solvent, which hinders the complete dissolution of polyphenolic compounds. Additionally, high ethanol concentrations may impede the dissolution of macromolecules, clogging the macropores of the tissue and thus negatively affecting polyphenol extraction [39]. The CPA yield increased with the extraction temperature from 40 °C and reaching its maximum at 70 °C (5.09 ± 0.23%), but it began to decline when the temperature exceeded 70 °C (Figure 1B). This is likely due to the fact that higher temperatures enhance the movement of solvent and solute molecules, promoting diffusion and thereby improving the extraction yield. The susceptibility of polyphenols to oxidation at high temperatures, leading to their conversion into non-polyphenolic derivatives [40]. The extraction time also affects the extraction yield of CPA, which significantly increased with extraction time and reached its maximum (5.45 ± 0.13%) at 75 min. However, when the extraction time exceeded 75 min, the polyphenol extraction yield began to decline (Figure 1C). The study on the *Vernonia cinerea* leaves found that the extraction yield of polyphenols was higher (7.28 ± 0.55%) at 2 h of concentration time than that 1 h, 3 h and 4 h [38]. This is likely due to the extended extraction time damaging the molecular structure of the polyphenols, leading to their oxidative degradation [41] (Khadaroo et al., 2020). The extraction yield of CPA increased with the increase of the solid-to-liquid ratio, and reached its maximum (4.81 ± 0.17%) at 1:20 g/mL. However, the extraction yield of CPA began to decline when the solid-to-liquid ratio exceeded 1:20 g/mL (Figure 1D). This may be because the increased solvent volume enhanced the contact area between the *A. dracunculus* powder and the solvent, within the range of 1:10 to 1:20 g/mL, allowing for more thorough dissolution of the polyphenols and a significant improvement in extraction efficiency. Conversely, when the solid-to-liquid ratio surpassed 1:20 g/mL, the increased solvent volume facilitated the dissolution of other impurities, thereby reducing the solubility of CPA [42].

### 3.2. Results of Response Surface Test

The experimental design employed a Box-Behnken methodology to investigate the synergistic effects among solvent concentrations, thermal extraction conditions, and phase ratio parameters. A total of seventeen systematically designed experimental configurations were implemented, with corresponding response data presented in Table 3. After analysis and fitting with Design-expert 13, the multiple quadratic regression equation was obtained as follows:

Y = +5.05 − 0.0025*A* + 0.049*B* + 0.051*C* + 0.0075*AB* + 0.097*AC* − 0.025*BC* − 0.64*A*^2^ − 0.81*B*^2^ − 0.38*C*^2^.

The predictive capability of the regression model was statistically validated through variance decomposition analysis, revealing significant concordance between theoretical predictions and experimental observations. As shown in Table 4, the model exhibited highly significant differences (*p* < 0.0001), with the lack-of-fit term yielding a *p* of 0.3237 (>0.05). The regression coefficient of R^2^ = 0.9984 indicated that the model had an excellent fit and could be used to analyze and predict the extraction process parameters of CPA. Factors *B*, *C*, *AC*, *A*^2^, *B*^2^, and *C*^2^ had an extremely significant impact on the extraction yield of CPA (*p* < 0.01), while factors *A, AB*, and BC showed no significant influence (*p* > 0.05). Based on the ANOVA results, the order of influence of the three factors on the response value was determined to be *C* > *B* > *A*.

**Figure 2:**
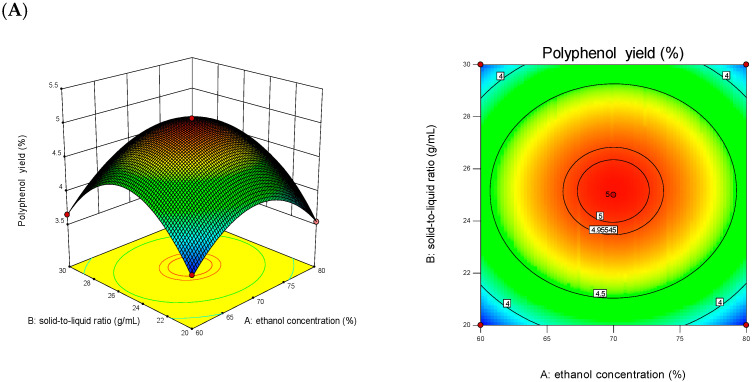

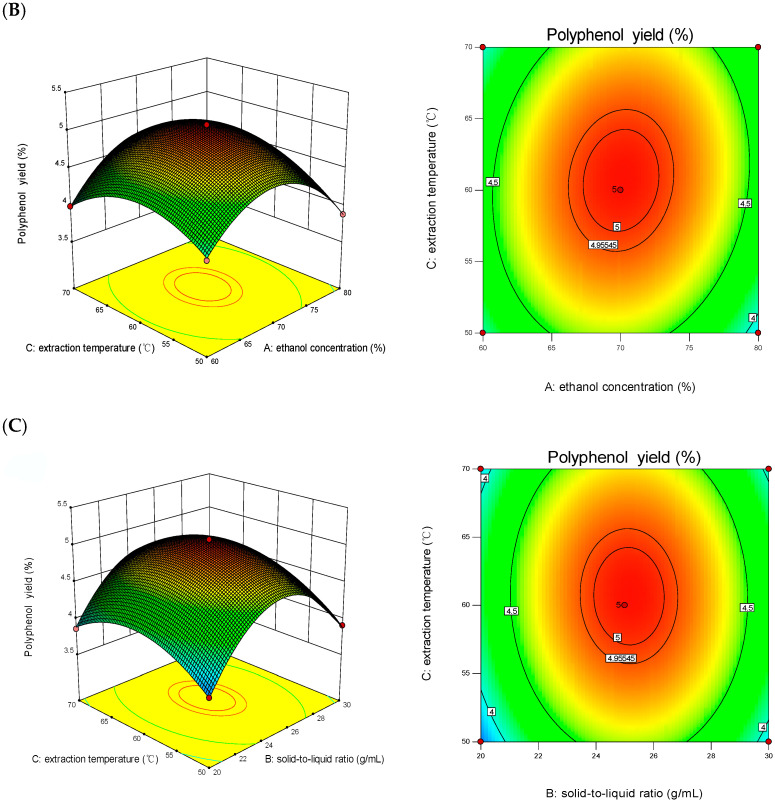
Response surface and contour diagram of the interaction of various factors on the yield of CPA. (**A**): ethanol concentration and solid-to-liquid ratio; (**B**): ethanol concentration and extraction temperature; (**C**): solid-to-liquid ratio and extraction temperature.

The response surface methodology based on Box-Behnken design enabled the establishment of a three-dimensional mathematical model illustrating the correlation between process parameters and extraction yield of CPA. Through this analytical approach, the gradient of the response surface curvature reflected the sensitivity of the target product yield to parameter variations, with steeper slopes indicating higher sensitivity and gentler slopes representing reduced factor influence. Furthermore, the geometric characteristics of contour plots provided valuable insights into factor interactions—circular patterns denoted negligible interaction effects, while elliptical distributions signified statistically significant interactions between variables [43]. The slope of the response surface is relatively gentle in Figure 2A, and the center of the AB interaction was approximately circular, suggesting a negligible interactive effect between ethanol concentration and the solid-to-liquid ratio. As illustrated in Figure 2B, the response surface analysis revealed a pronounced curvature gradient, accompanied by elliptical contour patterns at the AC interaction region, demonstrating a strong synergistic effect between ethanol concentration and the solid-to-liquid phase ratio. The three-dimensional response surface plot in Figure 2C demonstrated the combined influence of thermal conditions and phase ratio on phenolic compound recovery, exhibiting comparable characteristics to those observed in Figure 2A. This similarity in response patterns revealed minimal interactive effects between thermal extraction parameters and the solid-to-liquid proportion. Through systematic optimization using Design-Expert software13.0 (Stat-Ease Inc., Minneapolis, MN, USA)), the ideal operational conditions for CPA extraction were determined to be as follows: an ethanol concentration of 70.029% (*v*/*v*), solid-liquid ratio at 1:25.146 (g/mL), and extraction temperature at 60.676 °C. The predicted extraction yield of polyphenols was 5.048 ± 0.02%. Based on the predicted data, the optimal process parameters were adjusted to an ethanol concentration of 70%, solid–liquid ratio at 1:25 (g/mL), and extraction temperature at 60 °C. Under these conditions, the actual extraction yield of polyphenols was measured to be 5.02 ± 0.04%, which aligns well with the predicted value, demonstrating the feasibility of this method.

### 3.3. Purification of CPA by Macroporous Resin

Macroporous resins rely on Van der Waals forces or hydrogen bonding for adsorption, combining both adsorption and sieving processes. Their porous structure allows for the selective separation of particles based on size. The differences in adsorption and desorption capacities of macroporous resins for polyphenols are primarily influenced by factors such as polarity, specific surface area, and pore size. The choice of macroporous adsorbent material should prioritize several key performance indicators, including superior uptake capability, rapid kinetics in both absorption and release processes, and excellent efficiency in substance capture and recovery cycles [44].

In this experiment, five types of macroporous resins as NKA-9, D301, D101, AB-8 and D201 were evaluated through static adsorption and desorption tests. The adsorption and desorption rate was significantly different among different resins (*p* < 0.05) (Table 5). The D301 resin and D101 resin demonstrated a relatively high adsorption rate, at 64.3 ± 2.41% and 58.7 ± 2.06%, respectively. D101, D201, D301and NKA-9 are made from styrene-divinylbenzene (ST-DVB) and have larger surface areas compared to the AB-8 and NKA-9, enhancing interactions between resin and polyphenol compounds, resulting in more efficient adsorption [45]. Additionally, high adsorption capacity for the resins with large surface areas has also been reported for polyphenolic compounds from *Chaenomeles speciosa* (Sweet) Nakai fruit [46] and red onion (*Allium cepa* L.). Aliya [45] studied the purification of the polyphenols from red onion (*Allium cepa* L.) peel and found that XAD7HP showed the highest adsorption ratio (85.00%) and desorption ratio (87.10%) at the condition of pH 4, 25 °C, 7 h for adsorption, followed by desorption with 70% ethanol for 1 h at 25 °C. The polarity of the resins also affected the adsorption rate. D101 and NKA-9 are non-polar resins, D301 is a weak polar resin and D201 is a strong polar resin. In this study, the adsorption rate was higher at the weak (D301) and non-polar resin (D101). Xu [47] reported that non-polar or weakly polar resins exhibited a high adsorption capacity and a high desorption rate.

Meanwhile, the desorption rate of D101 (66.8 ± 3.17%) was higher than that of D201 (40.4 ± 2.13%), D301 (42.9 ± 1.77%), AB-8 (63.4 ± 3.42%) and NKA-9 (63.9 ± 3.57%). This trend is in line with the previous study by Hou and Zhang [48] which reported that resins with a larger pore size demonstrated superior desorption capacity. The desorption rate of the polyphenol compound is proportional to the resin pore size, which could be explained by the larger pore size facilitating easier elution of the adsorbate by the desorption agent [48]. Considering both the adsorption rate and the desorption rate, the D101 resin was selected for the purification of CPA.

### 3.4. Dynamic Adsorption and Desorption

**Figure 3:**
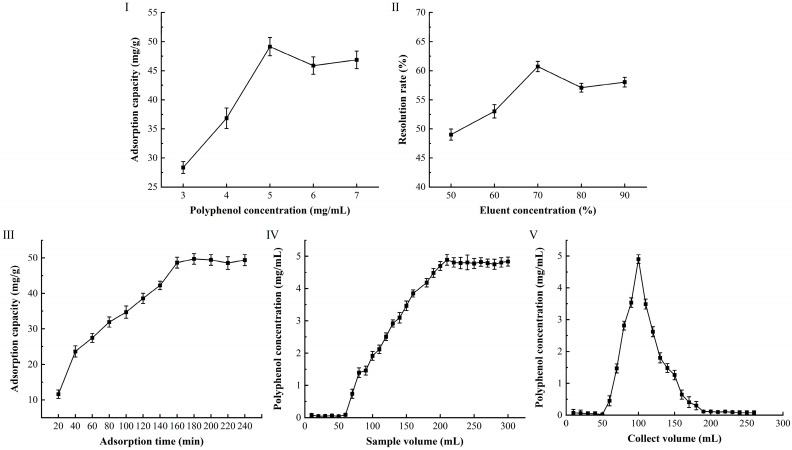
Adsorption efficiency based on the loading concentration of CPA (**I**); desorption efficiency based on eluent concentration (**II**); desorption efficiency based on eluent concentration (**III**); dynamic adsorption for CPA (**IV**); dynamic desorption for CPA (**V**).

The adsorption capacity of the macroporous resin increased with the concentration of CPA from 3mg/mL to 5mg/mL (Figure 3I) because the contact between phenolic compounds and the resin were enhanced by higher sample concentrations. At 5.0 mg/mL CPA concentration, the adsorption capacity of D101 macroporous resin for CPA stabilized, indicating that the resin’s adsorption capacity had reached saturation. Therefore, 5.0 mg/mL of CPA concentration was selected as the loading concentration. Among different ethanol concentrations, 70% ethanol could elute polyphenols from the resin, and the elution peak was relatively concentrated, with no trailing phenomenon (Figure 3II), this result was the same as Wu [44]. The adsorption of CPA by D101 macroporous resin occurred over a time range of 20 to 240 min (Figure 3III), in which, at the cut-off time, the polyphenols adsorption had reached equilibrium.

A 5 mg/mL of CPA extract was selected and loaded at a flow rate of 2.0 mL/min. The eluate was collected at 10 mL per tube using a fraction collector, and the polyphenol concentration in each tube was measured to plot the dynamic adsorption curve of the macroporous resin (Figure 3Ⅳ). The results showed that phenolics began to be detected in the eluate when the loading volume of the CPA extract reached 70 mL. The polyphenol content in the eluate gradually increased with the increase of the concentration of CPA extracts. When the loading volume reached 210 mL, the eluate concentration reached 4.89 ± 0.21 mg/mL, which was close to the loading concentration, indicating that the D101 resin had reached saturation.

The desorption experiment was conducted after the D101 macroporous resin reached adsorption saturation at 5 mg/mL of CPA extract (Figure 3V). A total of 260 mL of 70% ethanol was used for desorption of polyphenols at the flow rate of 1.8 mL/min. The polyphenols were detected until the desorption volume of 70% ethanol was about 60 mL, and about 80% of the polyphenols had been eluted by 70% ethanol when the desorption volume was about 190 mL. The eluate from 60 mL to 190 mL of desorption volume was combined, concentrated, and freeze-dried to obtain PPA. The PPA was 60.48 ± 1.87%, which was 4.57 times higher than that of CPA (13.23 ± 0.36%) as determined by the Folin–Ciocalteu method. Ren [25] studied the purification methods of wampee polyphenols (WPP) and found that AB-8 resin increased purity of (WPP) from 12.51% ± 0.36–53.8% ± 1.87%. This indicated that the polyphenols from different plant species need different macroporous resin to purify and that D101 macroporous resin with this method was suitable for the purification of *A. dracunculus* polyphenols.

### 3.5. Component Analysis of PPA by UPLC-QE

The study employed mass spectrometry targeted quantification, determining peaks based on the accurate mass-to-charge ratio and retention time of standards. The relative quantification of 36 polyphenols in artemisia annua polyphenols was performed using the signal intensity (peak area) of characteristic fragments or deprotonated molecules. This study employed the standard curve method, utilizing peak area for qualitative and quantitative analysis. All standard curves exhibited correlation coefficients greater than 0.995, indicating excellent linearity of the equations and the feasibility of the method. A total of 36 polyphenols were identified for PPA, which comprised 13 phenolic acids, 17 flavonoids, 3 benzaldehyde derivatives (3, 11, 13), 1 aromatic acid (5), 1 dihydrochalcone (32) and 1 stilbenes (26). The 13 phenolic acids consisted of 7 benzoic acid derivatives (1, 2, 4, 7, 9, 19, 23) and 6 cinnamic acid derivatives (8, 12, 16, 17, 29, 30). Among the 17 flavonoids, 4 flavonoids (6, 10, 22, 25), 5 flavonols (18, 24, 28, 35, 36), 2 flavanones (31, 34), 4 flavones (15, 20, 27, 33), and 2 flavonoid glycosides (14, 21) were identified.

**Figure 4:**
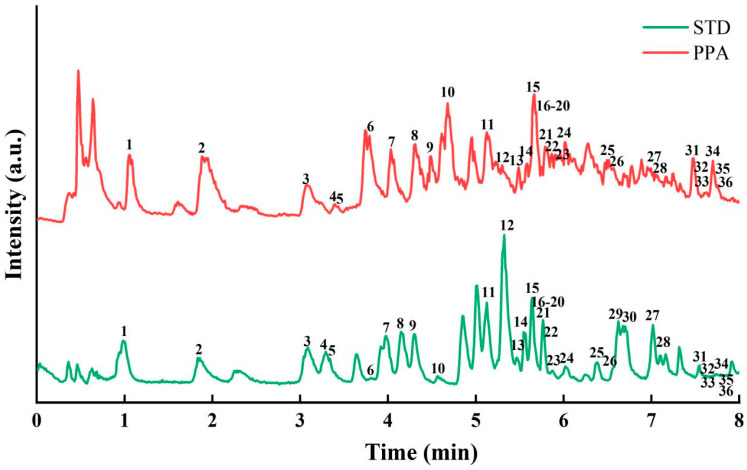
Chromatogram of STD (40 polyphenol standards) and PPA (purified polyphenol of *A. dracunculus*).1: Gallic acid; 2: Protocatechuic acid; 3: Protocatechualdehyde; 4: 4-Hydroxybenzoic acid; 5: Phthalic acid; 6: Catechin; 7: Vanillic acid; 8: Caffeic acid; 9: Syringic acid; 10: Epicatechin; 11: Vanillin; 12: Hydroxycinnamic Acid; 13: Syringaldehyde; 14: Rutin; 15: Vitexin; 16: Trans-Ferulic acid; 17: Sinapic Acid; 18: Quercetin 3-β-D-glucoside; 19: Salicylic acid; 20: Luteoloside; 21: Genistin; 22: (+)-Dihydroquercetin; 23: Benzoic acid; 24: Kaempferol-3-O-glucoside; 25: (+)-Dihydrokaempferol; 26: Resveratrol; 27: Luteolin; 28: Quercetin; 29: Hydrocinnamic acid; 30: Trans-Cinnamic acid; 31: Naringenin Chalcone; 32: Phloretin; 33: Apigenin; 34: Naringenin; 35: Kaempferol; 36: Isorhamnetin.

The polyphenol compounds of *A. dracunculus* were identified by using UPLC-QE with standard curves (Figure 4) and also many unidentified peaks with substantial abundance were observed in the PPA, highlighting the compositional diversity and structural complexity of PPA [49].

Table 6 shows the quantitative analysis of 36 common polyphenol compounds from PPA, with a total content of 5680.44 ng/mg. The highest polyphenol content was protocatechuic acid at 1338.05 ± 1.83 ng/mg, followed by Rutin at 884.39 ± 2.21 ng/mg, and Quercetin 3-β-D-glucoside at 868.29 ± 1.33 ng/mg. Protocatechuic acid is known for its broad-spectrum physiological activities, including cardiovascular protection, anti-hyperglycemic, anti-inflammatory, and antioxidant effects [50]. Rutin has pharmacological properties including anti-cancer, anti-inflammatory, neuroprotective, anti-proliferative and antioxidant stress activities [51]. Quercetin 3-β-D-glucoside also demonstrates significant therapeutic potential in the prevention and treatment of various chronic diseases [52], which indirectly suggests that PPA may possess these potential functionalities.

### 3.6. Infrared Spectroscopy (IR) Analysis

**Figure 5:**
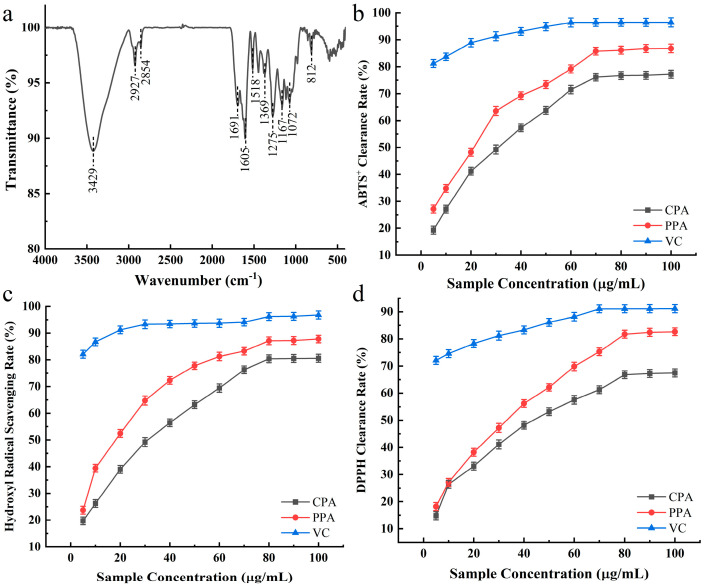
Infrared spectrum of PPA (**a**); DPPH radical scavenging assay (**b**); hydroxyl radical scavenging assay (**c**); ABTS^+^radical scavenging assay (**d**); Vc represented ascorbic acid.

As shown in Figure 5a, the absorption peak at 3429 cm^−1^ was typically associated with O-H stretching vibrations, indicating the presence of phenolic hydroxyl groups and suggesting the existence of phenolic compounds. The absorption peaks at 2927 cm^−1^ and 2854 cm^−1^ were generally attributed to C-H stretching vibrations, indicating the presence of methylene groups in the compound. The absorption peak near 1691 cm^−1^, corresponding to the carbonyl (C=O) stretching vibration, indicated the presence of phenolic acid compounds in PPA. The absorption peaks at 1605 cm^−1^ and 1518 cm^−1^, attributed to the C=C stretching vibrations of aromatic rings, indicated the presence of flavonoid compounds in PPA. Additionally, the C-O stretching vibrations observed in the range of 1275 cm^−1^ to 1369 cm^−1^ indicated the presence of tannin compounds in PPA. Additionally, the absorption peaks at 1167 cm^−1^ and 812 cm^−1^ indicated the potential presence of aromatic ring structures and associated C-O bonds in the sample. The analysis of these absorption peaks helped to identify the chemical structures and functional groups present in the sample [53].

### 3.7. In Vitro Determination of Antioxidant Properties

As shown in Figure 5b–d, VC, PPA, and CPA all demonstrated significant DPPH radical scavenging effects, hydroxyl radical scavenging effects and ABTS^+^ radical scavenging effects, and the scavenging capacities increased with the increasing of sample concentrations. At the same sample concentration, both CPA and PPA exhibited lower scavenging capacities compared to VC, with the overall scavenging effectiveness following the order Vc > PPA > CPA.

At a sample concentration of 80 μg/mL, the DPPH radical scavenging activity and hydroxyl radical scavenging activity reached to 81.73 ± 1.43% and 87.11 ± 1.57% for PPA, 66.81 ± 1.56% and 80.4 ± 1.54% for CPA, respectively. For the ABTS^+^ radical scavenging, the scavenging activity of PPA and CPA was 85.74 ± 1.52% and 76.1 ± 1.47% at a concentration of 70 μg/mL. These findings suggest that PPA has a superior radical scavenging capability. This corresponds to the results of Wang [54] and Aliya [45]; polyphenols exhibited concentration-dependent DPPH radical scavenging activity and ABTS•⁺ scavenging activity. The DPPH radical scavenging activity and ABTS scavenging activity of purified polyphenol of red onion (*Allium cepa* L.) peel reached 90.60 ± 1.40% and 98.75 ± 0.46% at 500 µg/mL, while for the crude polyphenol it was 18.50 ± 1.10% and 31.39 ± 0.46%, and the purified polyphenol has significantly higher antioxidant activity compared to the crude polyphenol [45]. This shows that purification could improve the antioxidant activity of polyphenols.

### 3.8. Determination of Antibacterial and Antifungal Activity

The antibacterial activity of PPA against ten different pathogenic bacteria is shown in Table 7. PPA exhibited significant antibacterial activity against nine pathogenic bacteria, with the exception of *E. coli.* The diameter of the inhibition zones gradually expanded as the PPA concentration increased. Among the ten pathogenic bacteria, the PPA exhibited the most significant antibacterial effects against *S. aureus*, *B. subtilis*, and *R. stolonifer*, and the inhibition zone diameters reached 17.23 ± 0.21 mm, 18.47 ± 0.34 mm, and 18.61 ± 0.25 mm, at a concentration of 150 mg/mL, respectively. At lower PPA concentrations, the inhibition zone diameters of *S. flexneri*, *E. faecalis*, *L. monocytogenes*, *A. baumannii* and *P. aeruginosa* were relatively small, and the inhibition zone diameters of these five pathogenic bacteria at 25 mg/mL were at a concentration lower than 7.12 ± 0.07 mm. The antibacterial activity progressively intensified with the increased of PPA concentrations, the inhibition zone diameters reached the highest at 150 mg/mL of concentration, at 11.64 ± 0.16 mm, 15.97 ± 0.13 mm, 9.95 ± 0.24 mm, 10.27 ± 0.16 mm and 12.37 ± 0.27 mm, respectively.

PPA had no antibacterial activity against *S. enterica* at 25 mg/mL of concentration and exhibited weak antibacterial activity at 50 mg/mL of concentration with a diameter of only 7.41 ± 0.21 mm. However, there was no significant difference in the diameter of the inhibition zones between the concentration of 150 mg/mL and 200 mg/mL, for the nine pathogenic bacteria except *E. coli*., which showed no inhibition zones at any concentration tested, indicating that PPA has no inhibitory effect on *E. coli*.

As shown in Table 7, the polyphenols demonstrated the strongest inhibitory effects against *S. aureus*, *B. subtilis* and *R. stolonifer*, with a minimum inhibitory concentration (MIC) of 3.125 mg/mL for *S. aureus*, *B. subtilis* and 6.25 mg/mL for *R. stolonifer*. Additionally, the polyphenols showed notable antibacterial activity against *S. flexneri* and *P. aeruginosa*, with an MIC value of 6.25 mg/mL. However, the PPA inhibitory effects against *L. monocytogenes* and *A. baumannii* was relatively weaker, with an MIC value of 12.5 mg/mL. The polyphenols exhibited growth inhibition against *S. flexneri* and *E. faecalis* with the MIC value of 25 mg/mL. Xu [55] investigated the antibacterial properties of the polyphenol of *Smilax china* L. and reported that it exhibited antibacterial activity against five bacteria (*Salmonella typhimurium*, *Listeria monocytogenes*, *Staphylococcus aureus*, *Bacillus subtilis*, and *Escherichia coli*). The MIC values of the polyphenol of *S. china* against *S. typhimurium*, *L. monocytogenes* and *B. subtilis* were 781.25 μg/mL, while *S. aureus* and *E. coli* were more sensitive to SCLP with a MIC value of 195.31 μg/mL. Zurek [30] studied the antibacterial and antifungal effects of polyphenolic fraction of *Juglans regia* L.; the results showed that *J. regia* has a bactericidal effect against the microorganisms in the order as *Klebsiella pneumoniae* > *S. aureus* > *Pseudomonas aeruginosa* > *Streptococcus pyogenes* > *E. coli* > *Enterococcus faecalis* at the concentration of 10 mg/mL. Our results are consistent with other findings indicating that polyphenol extracts are effective agents against many bacterial species [56].

In terms of bactericidal activity, the polyphenols showed the strongest effect against *B. subtilis*, with MBC of 3.125 mg/mL. The MBC values for *S. aureus* and *R. stolonifer* were 6.25 mg/mL. For *S. enterica* and *P. aeruginosa*, the MBC value was 25 mg/mL. The polyphenols demonstrated bactericidal activity against *E. faecalis*, *L. monocytogenes*, *A. baumannii*, and *S. enterica* at the highest concentration tested, with MBC of 25 mg/mL and 50 mg/mL.

Notably, the MIC and MBC values of the polyphenols against *E. coli.* were both 0 mg/mL, indicating no antibacterial or bactericidal activity against this bacterium. The results from the two testing methods were largely consistent, and this result was also reported by Jovanović [57]. This result showed that the antibacterial activity of PPA was higher for Gram-positive bacteria than that of Gram-negative bacteria. This corresponds with the results of Martinenghi [58], in which cannabinoids from *Cannabis sativa* also showed a significant antimicrobial effect on the Gram-positive as *S. aureus* and *S. taphylococcus* epidermidis, while no activity was noticed on Gram-negative *E. coli*. These findings hold significant implications for the further development of antimicrobial research, as the prevalence of drug-resistant microorganisms in food and clinical environments continues to rise [59]. The ten selected pathogenic bacteria are all harmful to humans or animals, and thus this study highlights the potential of *A. dracunculus* as a natural, safe, and effective food preservative.

## 4. Conclusions

This study established an efficient extraction and purification protocol for polyphenols from *A. dracunculus* (PPA), yielding a 5.02% extraction rate under optimized conditions (70% ethanol, 70 °C, 1:20 solid–liquid ratio). Purification with D101 macroporous resin enhanced PPA purity to 60.48% (4.57-fold higher than crude extract), with protocatechuic acid (1338.05 ng/mg) identified as the dominant compound among 36 polyphenols via UPLC-QE. FT-IR confirmed phenolic acids, flavonoids, and tannins in PPA. PPA exhibited dose-dependent scavenging of DPPH (81.73%), hydroxyl (87.11%), and ABTS+ (85.74%) radicals at 70–80 μg/mL. PPA also inhibited 9/10 pathogens, notably *S. aureus*, *B. subtilis* (MIC: 3.125 mg/mL), and *R. stolonifer* (MIC: 6.25 mg/mL). The PPA exhibited strong antioxidant and antibacterial activities in vitro, suggesting their potential as antioxidants and antibacterial agents in functional foods and pharmaceutical applications. This study contributes to the better utilization and development of *Artemisia dracunculus* resources, uncovering their potential value for applications in fields such as food and medicine. However, the solvent extraction method in this study exhibits low extraction efficiency and struggles to achieve selective extraction of specific polyphenolic components. In addition, this study primarily evaluated antioxidant and antibacterial activities in vitro, which may not fully translate to in vivo efficacy. The specific bioactive compounds responsible for the observed effects and their molecular mechanisms remain to be elucidated. Therefore, further investigation is needed to improve the efficiency and selectivity for target polyphenols by using advanced extraction techniques such as ultrasound-assisted extraction, supercritical CO_2_ extraction, and the study of the bioavailability of PPA in animal models along with the employment of omics technologies such as metabolomics and proteomics to identify key bioactive compounds and their mode of action.

## Figures and Tables

**Table 1:** Physical properties of the five types of macroporous resins assessed in this study.

Resins	D101	D201	D301	AB-8	NKA-9
Physicochemical properties					
Color	Milky white	Light yellow	Milky white	Milky white	Milky white
Particle size range (mm)	0.9–1.0	0.5–0.7	0.4–0.6	1.3–1.4	1.5–1.65
Polarity	Non-polarity	Strong-polarity	Weakly-polarity	Middle-polarity	Non-polarity
Water content (%)	65–75	65–75	65–75	60–70	65–75
Specific surface area (m^2^/g)	500–550	650–700	450–480	480–520	250–290

**Table 2:** Independent variables and their levels used for Box-Behnken design (BBD).

Independent Variables	Coded Levels of Variables		
	−1	0	1
Ethanol concentration (A) (%)	60	70	80
Solid-to-liquid ratio (B) (g/mL)	1:20	1:25	1:30
Extraction temperature (C) (°C)	50	60	70

**Table 3:** Results of response surface test.

No.	A	B	C	Polyphenol Yield (%)	
	Ethanol Concentration (%)	Solid-to-Liquid Ratio (g/mL)	Extraction Temperature (°C)	Actual Value	Predicted Value
1	60	20	60	3.61	3.59
2	80	20	60	3.57	3.54
3	60	30	60	3.62	3.65
4	80	30	60	3.61	3.63
5	60	25	50	4.07	4.05
6	80	25	50	3.84	3.88
7	60	25	70	4.02	3.99
8	80	25	70	4.26	4.21
9	70	20	50	3.79	3.74
10	70	30	50	3.88	3.91
11	70	20	70	3.91	3.86
12	70	30	70	3.87	3.93
13	70	25	60	4.94	5.00
14	70	25	60	5.06	5.05
15	70	25	60	5.09	5.07
16	70	25	60	5.13	5.08
17	70	25	60	5.00	5.03

**Table 4:** Evaluation of regression equation model coefficients and their significance test.

Source	Square Sum	Degrees of Freedom	Mean Square Sum	*F*-Value	*p*-Value
model	5.66	9	0.6289	486.70	0.0001 **
*A*-Ethanol concentration	0.0000	1	0.0000	0.0387	0.8496
*B*-Solid-to-liquid ratio	0.0190	1	0.0190	14.71	0.0064 **
*C*-Extraction temperature	0.0210	1	0.0210	16.26	0.0050 **
*AB*	0.0002	1	0.0002	0.1741	0.6890
*AC*	0.0380	1	0.0380	29.43	0.0010 **
*BC*	0.0025	1	0.0025	1.93	0.2068
*A* ^2^	1.70	1	1.70	1316.00	0.0001 **
*B* ^2^	2.75	1	2.75	2127.40	0.0001 **
*C* ^2^	0.6016	1	0.6016	465.60	0.0001 **
residual	0.0090	7	0.0013		
lost proposal	0.0049	3	0.0016	1.59	0.3237
pure error	0.0041	4	0.0010		
aggregate	5.67	16			
R^2^ = 0.9984		Adj R^2^ = 0.9964		C.V% = 0.8581	

Note: ** *p* < 0.01; the difference is highly significant.

**Table 5:** Adsorption and desorption results of CPA on different resins.

	D101	D201	D301	AB-8	NKA-9
Adsorption Rate (%)	58.7 ± 2.06 ^b^	43.3 ± 1.89 ^c^	64.3 ± 3.41 ^a^	38.7 ± 1.88 ^d^	36.7 ± 1.94 ^d^
Desorption Rate (%)	66.8 ± 3.17 ^a^	40.4 ± 2.13 ^c^	42.9 ± 1.77 ^c^	63.4 ± 3.42 ^b^	63.9 ± 3.57 ^b^

Different lowercase letters in each line denote statistically significant differences of adsorption rate and desorption rate between different resins, as determined by Duncan’s multiple range test.

**Table 6:** Composition of PPA.

Classification		No.	Name	RT (min)	MM	[M-H]-	MS/MS	Formula	Chemical Structure	Content ng/mg
phenolic acids	benzoic acid derivatives	1	Gallic acid	0.98	170.13	169.01	69.03/79.01/107.01/125.02	C_7_H_6_O_5_	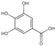	2.07 ± 0.11
		2	Protocatechuic acid	1.86	154.13	153.02	81.03/91.02/108.02/109.03	C_7_H_6_O_4_	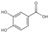	1338.05 ± 1.83
		4	4-Hydroxybenzoic acid	3.41	138.13	137.02	65.04/92.03/93.03	C_7_H_6_O_3_	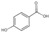	82.95 ± 0.15
		7	Vanillic acid	4.16	168.16	167.03	95.01/108.02/123.01152.01	C_8_H_8_O_4_	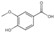	33.48 ± 0.07
		9	Syringic acid	4.49	198.19	197.05	125.02/138.03/153.02/182.02	C_9_H_10_O_5_	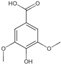	13.28 ± 0.16
		19	Salicylic acid	5.69	138.13	137.02	65.04/92.02/93.03	C_7_H_6_O_3_	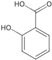	0.95 ± 0.01
		23	Benzoic acid	5.99	122.13	121.03	65.04/77.04/93.03	C_7_H_6_O_2_	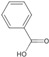	5.85 ± 0.03
	cinnamic acid derivatives	8	Caffeic acid	4.34	180.17	179.03	93.03/107.01/134.04/135.05	C_9_H_8_O_4_	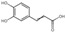	308.12 ± 0.18
		12	Hydroxycinnamic Acid	5.30	164.17	163.04	65.04/93.03/19.05	C_9_H_8_O_3_	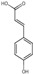	20.09 ± 0.05
		16	Trans-Ferulic acid	5.63	194.20	193.05	117.03/134.04/149.02/178.03	C_10_H_10_O_4_	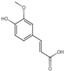	5.45 ± 0.04
		17	Sinapic Acid	5.65	224.23	223.06	149.02/164.01/179.04/208.04	C_11_H_12_O_5_	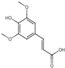	1.14 ± 0.02
		29	Hydrocinnamic acid	7.10	150.19	149.06	77.04//91.05/105.03	C_9_H_10_O_2_	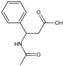	0.07 ± 0.00
		30	Trans-Cinnamic acid	7.19	148.17	147.04	77.04/102.05/103.05	C_9_H_8_O_2_	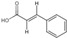	0.47 ± 0.01
flavonoids	flavonoids	6	Catechin	3.98	290.29	289.07	125.02/179.03/203.07/245.08	C_15_H_14_O_6_	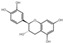	524.33 ± 0.15
		10	Epicatechin	4.80	290.29	29.07	125.02/	C_15_H_14_O_6_	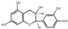	70.13 ± 0.15
		22	(+)-Dihydroquercetin	5.89	304.27	303.05	125.02/217.05/257.05/285.04	C_15_H_12_O_7_	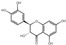	269.94 ± 0.37
		25	(+)-Dihydrokaempferol	6.41	288.27	287.06	151.00/213.06/241.05/269.05	C_15_H_12_O_6_	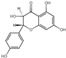	14.69 ± 0.09
	flavonols	18	Quercetin 3-β-D-glucoside	5.69	464.41	463.09	255.03/271.04/301.04/300.03	C_21_H_20_O_12_	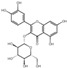	868.29 ± 1.33
		24	Kaempferol- 3-O-glucoside	6.01	448.41	447.09	227.04/255.03/284.03/285.04	C_21_H_20_O_11_	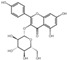	759.22 ± 0.27
		28	Quercetin	7.05	302.25	313.03	107.01/151.00/229.05/257.05/273.04	C_15_H_10_O_7_	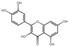	64.62 ± 0.19
		35	Kaempferol	7.63	286.25	285.04	107.01/151.00/227.04/255.03	C_15_H_10_O_6_	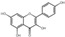	68.05 ± 0.67
		36	Isorhamnetin	7.68	316.28	315.05	151.00/255.03/271.03/300.03	C_16_H_12_O_7_	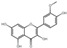	29.37 ± 0.48
	flavones	15	Vitexin	5.61	432.41	431.10	161.02/269.05/283.06/311.06	C_21_H_20_O_10_	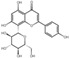	1.31 ± 0.06
		20	Luteoloside	5.72	448.41	447.09	151.00/257.05/284.03/285.04	C_21_H_20_O_11_	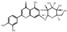	31.73 ± 0.26
		27	Luteolin	7.01	286.25	285.04	151.00/175.04/199.04/217.05	C_15_H_10_O_6_	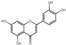	125.49 ± 0.43
		33	Apigenin	7.53	270.25	269.05	117.03/151.00/201.06/225.06	C_15_H_10_O_5_	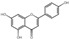	36.10 ± 0.10
	flavanones	34	Naringenin	7.54	272.27	271.06	107.01/119.05/177.06/151.00	C_15_H_12_O_5_	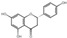	6.50 ± 0.09
		31	Naringenin Chalcone	7.49	272.27	271.06	107.01/151.04/177.06/227.07	C_15_H_12_O_5_	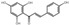	5.16 ± 0.17
	flavonoid Glycosides	14	Rutin	5.55	610.57	609.15	255.03/271.03/300.03/301.04	C_27_H_30_O_16_	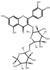	884.39 ± 2.21
		21	Genistin	5.78	432.41	431.10	133.03/225.06/268.04/269.05	C_21_H_20_O_10_	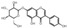	5.10 ± 0.19
dihydrochalcone		32	Phloretin	7.51	274.29	273.08	93.03/125.02/167.04	C_15_H_14_O_5_	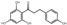	1.08 ± 0.04
benzaldehyde derivatives		3	Protocatechualdehyde	3.16	138.13	137.02	64.02/81.03/92.03/108.02	C_7_H_6_O_3_	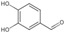	80.97 ± 0.03
		11	Vanillin	5.12	152.16	151.04	81.03/92.03/108.02/136.02	C_8_H_8_O_3_	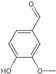	6.25 ± 0.12
		13	Syringaldehyde	5.44	182.19	181.05	108.02/137.02/151.04/166.03	C_9_H_10_O_4_	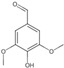	3.45 ± 0.10
stilbenes		26	Resveratrol	6.64	228.26	227.07	93.04/143.05/185.06	C_14_H_12_O_3_	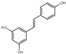	0.46 ± 0.01
other aromatic acid		5	Phthalic acid	3.49	166.14	165.02	76.02/93.03/121.03	C_8_H_6_O_4_	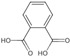	11.81 ± 0.31

**Table 7:** Antibacterial and antifungal activity for PPA.

Strain Name	25 mg/mL	50 mg/mL	75 mg/mL	100 mg/mL	125 mg/mL	150 mg/mL	200 mg/mL	MIC (mg/mL)	MBC (mg/mL)
	Siameter of the Inhibition Zone (mm)								
*Salmonella enterica*	-	7.41 ± 0.21	8.63 ± 0.17	9.41 ± 0.19	10.12 ± 0.23	11.31 ± 0.16	11.17 ± 0.14	25	50
*Staphylococcus aureus*	10.4 ± 0.17	12.3 ± 0.16	13.7 ± 0.23	14.4 ± 0.11	15.67 ± 0.14	17.23 ± 0.21	17.35 ± 0.17	3.125	6.25
*Bacillus subtilis*	10.6 ± 0.13	12.5 ± 0.13	13.9 ± 0.07	15.2 ± 0.12	16.31 ± 0.26	18.47 ± 0.34	18.36 ± 0.13	3.125	3.125
*Shigella flexneri*	7.12 ± 0.07	8.23 ± 0.12	9.7 ± 0.13	10.3 ± 0.06	11.76 ± 0.15	11.64 ± 0.16	11.81 ± 0.14	6.25	12.5
*Enterococcus faecalis*	7.89 ± 0.09	9.31 ± 0.11	12.2 ± 0.16	13.4 ± 0.14	14.38 ± 0.17	15.97 ± 0.13	15.37 ± 0.12	25	25
*Escherichia coli*	-	-	-	-	-	-	-	-	-
*Listeria monocytogenes*	7.17 ± 0.04	8.36 ± 0.06	9.39 ± 0.13	9.42 ± 0.21	9.57 ± 0.16	9.95 ± 0.24	9.88 ± 0.10	12.5	25
*Acinetobacter baumannii*	7.23 ± 0.06	8.47 ± 0.09	9.23 ± 0.11	9.36 ± 0.14	9.48 ± 0.13	10.27 ± 0.16	10.29 ± 0.13	12.5	25
*Pseudomonas aeruginosa*	7.87 ± 0.15	8.68 ± 0.14	9.42 ± 0.13	10.2 ± 0.11	11.34 ± 0.16	12.37 ± 0.27	12.88 ± 0.23	6.25	12.5
*Rhizopus stolonifer*	9.52 ± 0.12	12.3 ± 0.11	13.8 ± 0.16	15.6 ± 0.15	17.63 ± 0.13	18.61 ± 0.25	18.23 ± 0.32	6.25	6.25

## Data Availability

The data presented in this study are available on request from the corresponding author. The data are not publicly available due to privacy reasons.

## Abbreviations

The following abbreviations are used in this manuscript:

CPA	Crude polyphenols of *A. dracunculus*
PPA	Purified polyphenols of *A. dracunculus*
FT-IR	Fourier Transform Infrared Spectroscopy
UPLC-QE	Ultra-Performance Liquid Chromatography-Q Exactive
